# Supplementary material for: Phenological strategies of an evergreen tree in the Caatinga
Source: PLoS One. 2025 Mar 7;20(3):e0317522. doi: 10.1371/journal.pone.0317522 (PMC11888133; doi:10.1371/journal.pone.0317522)
Supplement: Attachment — Submitted filename: PONE-D-24-27053-vetted.pdf [file pone.0317522.s002.pdf]

PLOS ONE

Functional strategies of Sarcomphalus joazeiro in a Brazilian Seasonally Dry Tropical Forest Fragment

--Manuscript Draft--

**Manuscript Number:**	PONE-D-24-27053
**Article Type:**	Research Article
**Full Title:**	Functional strategies of Sarcomphalus joazeiro in a Brazilian Seasonally Dry Tropical Forest Fragment
**Short Title:**	Functional Strategies of Sarcomphalus joazeiro in Brazilian SDTF Fragment
**Corresponding Author:**	Fábio de Almeida Vieira<br>Universidade Federal do Rio Grande do Norte<br>Macaíba, RN, RN BRAZIL
**Keywords:**	juazeiro; forest ecology; phenology of tropical forests; seasonality
**Abstract:**	The Seasonally Dry Tropical Forests (SDTF) represent 40% of global tropical forests, predominantly found in the Neotropical region, especially in the Caatinga, the largest continuous formation of this type. This region is marked by threats such as deforestation and desertification, demanding urgent conservation efforts. This study aimed to investigate the ecological and drought tolerance strategies adopted by Sarcomphalus joazeiro (Mart.), a commercially valuable and ecologically important tree species in the Caatinga biome. Intrapopulational monitoring of vegetative and reproductive phenophases of trees was conducted in a forest fragment in Brazil. We analyzed the seasonality of phenophases, spatial distribution of reproductive synchrony, correlation with meteorological variables, and biometry of fruits and seeds. The results demonstrated that the flowering and fruiting of S. joazeiro is annual, with synchrony and flowering period observed as a reproductive strategy, exhibiting an aggregated spatial pattern. Sarcomphalus joazeiro is one of the few species that flowers during the dry season, potentially serving as an important food resource. The species show phenological performance with little correlation to meteorological variables. Therefore, the research contributes to understanding the complex dynamics of these forests, which is crucial for anticipating and mitigating the impacts of climate change in these vulnerable regions and promoting effective conservation strategies.
**Order of Authors:**	Fernanda Moura Fonseca Lucas
	Kyvia Pontes Teixeira das Chagas
	Ageu da Silva Monteiro Freire
	Vivian Raquel Bezerra de Sousa
	Fábio de Almeida Vieira
**Additional Information:**	
**Question**	**Response**
**Financial Disclosure**<br><br>Enter a financial disclosure statement that describes the sources of funding for the work included in this submission. Review the submission guidelines for detailed requirements. View published research articles from *PLOS ONE* for specific examples.<br><br>This statement is required for submission	Yes

Powered by Editorial Manager® and ProduXion Manager® from Aries Systems Corporation

and **will appear in the published article** if the submission is accepted. Please make sure it is accurate.<br><br>**Funded studies**<br>Enter a statement with the following details:<br>• Initials of the authors who received each award<br>• Grant numbers awarded to each author<br>• The full name of each funder<br>• URL of each funder website<br>• Did the sponsors or funders play any role in the study design, data collection and analysis, decision to publish, or preparation of the manuscript?<br><br>Did you receive funding for this work?	
Please add funding details.<br>as follow-up to "**Financial Disclosure**<br><br>Enter a financial disclosure statement that describes the sources of funding for the work included in this submission. Review the submission guidelines for detailed requirements. View published research articles from *PLOS ONE* for specific examples.<br><br>This statement is required for submission and **will appear in the published article** if the submission is accepted. Please make sure it is accurate.<br><br>**Funded studies**<br>Enter a statement with the following details:<br>• Initials of the authors who received each award<br>• Grant numbers awarded to each author<br>• The full name of each funder<br>• URL of each funder website<br>• Did the sponsors or funders play any role in the study design, data collection and analysis, decision to publish, or preparation of the manuscript?<br><br>Did you receive funding for this work?"	This work was financed in part by the Office to Coordinate Improvement of Higher Education Personnel - Brazil (CAPES) - Finance Code 001. The authors acknowledge the Conselho Nacional de Desenvolvimento Científico e Tecnológico (CNPq) for their financial support (Grant no. 407700/2023-4).

Please select the country of your main research funder (please select carefully as in some cases this is used in fee calculation).<br>as follow-up to "**Financial Disclosure**<br><br>Enter a financial disclosure statement that describes the sources of funding for the work included in this submission. Review the submission guidelines for detailed requirements. View published research articles from *PLOS ONE* for specific examples.<br><br>This statement is required for submission and **will appear in the published article** if the submission is accepted. Please make sure it is accurate.<br><br>**Funded studies**<br>Enter a statement with the following details:<br>• Initials of the authors who received each award<br>• Grant numbers awarded to each author<br>• The full name of each funder<br>• URL of each funder website<br>• Did the sponsors or funders play any role in the study design, data collection and analysis, decision to publish, or preparation of the manuscript?<br><br>Did you receive funding for this work?"	BRAZIL - BR
**Competing Interests**<br><br>Use the instructions below to enter a competing interest statement for this submission. On behalf of all authors, disclose any competing interests that could be perceived to bias this work—acknowledging all financial support and any other relevant financial or non-financial competing interests.<br><br>This statement is required for submission and **will appear in the published article** if the submission is accepted. Please make sure it is accurate and that any funding	NO authors have competing interests

sources listed in your Funding Information later in the submission form are also declared in your Financial Disclosure statement.<br><br>View published research articles from *PLOS ONE* for specific examples.<br><br>**NO authors have competing interests**<br><br>Enter: *The authors have declared that no competing interests exist.*<br><br>**Authors with competing interests**<br><br>Enter competing interest details beginning with this statement:<br><br>*I have read the journal's policy and the authors of this manuscript have the following competing interests: [insert competing interests here]*<br><br>* typeset	
**Ethics Statement**<br><br>Enter an ethics statement for this submission. This statement is required if the study involved:<br><br>• Human participants<br>• Human specimens or tissue<br>• Vertebrate animals or cephalopods<br>• Vertebrate embryos or tissues<br>• Field research<br><br>Write "N/A" if the submission does not require an ethics statement.<br><br>General guidance is provided below. Consult the submission guidelines for detailed instructions. **Make sure that all information entered here is included in the Methods section of the manuscript.**	N/A

**Format for specific study types**<br><br>**Human Subject Research (involving human participants and/or tissue)**<br>• Give the name of the institutional review board or ethics committee that approved the study<br>• Include the approval number and/or a statement indicating approval of this research<br>• Indicate the form of consent obtained (written/oral) or the reason that consent was not obtained (e.g. the data were analyzed anonymously)<br><br>**Animal Research (involving vertebrate animals, embryos or tissues)**<br>• Provide the name of the Institutional Animal Care and Use Committee (IACUC) or other relevant ethics board that reviewed the study protocol, and indicate whether they approved this research or granted a formal waiver of ethical approval<br>• Include an approval number if one was obtained<br>• If the study involved *non-human primates*, add *additional details* about animal welfare and steps taken to ameliorate suffering<br>• If anesthesia, euthanasia, or any kind of animal sacrifice is part of the study, include briefly which substances and/or methods were applied<br><br>**Field Research**<br><br>Include the following details if this study involves the collection of plant, animal, or other materials from a natural setting:<br>• Field permit number<br>• Name of the institution or relevant body that granted permission	
**Data Availability**<br><br>Authors are required to make all data underlying the findings described fully available, without restriction, and from the time of publication. PLOS allows rare exceptions to address legal and ethical concerns. See the PLOS Data Policy and FAQ for detailed information.	Yes - all data are fully available without restriction

Powered by Editorial Manager® and ProduXion Manager® from Aries Systems Corporation

A Data Availability Statement describing where the data can be found is required at submission. Your answers to this question constitute the Data Availability Statement and **will be published in the article**, if accepted.<br><br>**Important:** Stating 'data available on request from the author' is not sufficient. If your data are only available upon request, select 'No' for the first question and explain your exceptional situation in the text box.<br><br>Do the authors confirm that all data underlying the findings described in their manuscript are fully available without restriction?	
**Describe where the data may be found in full sentences. If you are copying our sample text, replace any instances of XXX with the appropriate details.**<br><br>• If the data are **held or will be held in a public repository**, include URLs, accession numbers or DOIs. If this information will only be available after acceptance, indicate this by ticking the box below. For example: *All XXX files are available from the XXX database (accession number(s) XXX, XXX.).*<br>• If the data are all contained **within the manuscript and/or Supporting Information files**, enter the following: *All relevant data are within the manuscript and its Supporting Information files.*<br>• If neither of these applies but you are able to provide **details of access elsewhere**, with or without limitations, please do so. For example:<br><br>*Data cannot be shared publicly because of [XXX]. Data are available from the XXX Institutional Data Access / Ethics Committee (contact via XXX) for researchers who meet the criteria for access to confidential data.*<br><br>*The data underlying the results presented in the study are available from (include the name of the third party*	All relevant data are within the manuscript and its Supporting Information files.

and contact information or URL).<br>• This text is appropriate if the data are owned by a third party and authors do not have permission to share the data.<br><br>* typeset	
Additional data availability information:	

Manuscript Click here to access/download;Manuscript;Manuscript_final.docx

Functional strategies of *Sarcomphalus joazeiro* in a Brazilian Seasonally Dry Tropical Forest Fragment

Fernanda Moura Fonseca Lucas[1], Kyvia Pontes Teixeira das Chagas[2], Ageu da Silva Monteiro Freire[2], Vivian Raquel Bezerra de Sousa[3], Fábio de Almeida Vieira[3]*

[1] Federal University of Espírito Santo - UFES, Department of Forestry and Wood Sciences, Avenue Governador Lindemberg, 316, 29550-000 Jeronimo Monteiro, ES, Brazil

[2] Federal University of Paraná - UFPR, Department of Forestry Sciences, Avenue Prefeito Lothário Meissner, 632 - Jardim Botânico, 80210-170 Curitiba, PR, Brazil

[3] Federal University of Rio Grande do Norte - UFRN, Academic Unit Specialized in Agricultural Sciences, Rodovia RN 160, Km 03, 59280-000 Macaíba, RN, Brazil

16-digit ORCID:

Lucas, F. M. F., fernanda-fonseca@hotmail.com, ORCID: 0000-0002-3181-2568

Chagas, K. P. T., kyviapontes@gmail.com, ORCID: 0000-0003-1361-3204

Freire, A. S. M., ageufreire@hotmail.com, ORCID: 0000-0002-5364-0859

Sousa, V. R. B., viviansousa2011@hotmail.com, ORCID: 0009-0005-2188-4102

*Vieira, F. A., fabio.almeida@ufrn.br (*Corresponding author*), ORCID: 0000-0003-3347-255X

Abstract

The Seasonally Dry Tropical Forests (SDTF) represent 40% of global tropical forests, predominantly found in the Neotropical region, especially in the Caatinga, the largest continuous formation of this type. This region is marked by threats such as deforestation and desertification, demanding urgent conservation efforts. This study aimed to investigate the ecological and drought tolerance strategies adopted by *Sarcomphalus joazeiro* (Mart.), a commercially valuable and ecologically important tree species in the Caatinga biome. Intrapopulational monitoring of vegetative and reproductive phenophases of trees was conducted in a forest fragment in Brazil. We analyzed the seasonality of phenophases, spatial distribution of reproductive synchrony, correlation with meteorological variables, and biometry of fruits and seeds. The results demonstrated that the flowering and fruiting of *S. joazeiro* is annual, with synchrony and flowering period observed as a reproductive strategy, exhibiting an aggregated spatial pattern. *Sarcomphalus joazeiro* is one of the few species that flowers during the dry season, potentially serving as an important food resource. The species show phenological performance with little correlation to meteorological variables. Therefore, the research contributes to understanding the complex dynamics of these forests, which is crucial for anticipating and mitigating the impacts of climate change in these vulnerable regions and promoting effective conservation strategies.

Keywords: juazeiro, forest ecology, phenology of tropical forests, seasonality

1. Introduction

The Seasonally Dry Tropical Forests (SDTF) comprise a vegetation type within a complex of typologies, considered a global metacommunity (Pennington et al., 2009). SDTFs occupy approximately 40% of the world's tropical forests and are primarily located in the Neotropical region (Murphy & Lugo, 2003). In Brazil, they are predominantly found in the Northeast region, within a nationally recognized biome known as the Caatinga.

The Caatinga is the largest continuous global formation of Seasonally Dry Tropical Forests (SDTF) (Queiroz et al., 2018), characterized by high diversity and composed of a flora exceeding 4,000 species, of which approximately 20% are endemic (Silva & Souza, 2018; Zappi et al., 2015). Its remarkable diversity is represented by a species/area ratio nearly twice that of the Amazon rainforest (Fernandes et al., 2022). Furthermore, the plant communities adapted to drought have resulted from a mosaic of evolutionary histories involving widespread and accumulated immigration from surrounding biogeographic regions, as well as recent events of in situ speciation (Fernandes et al., 2022).

Despite its ecological importance, the Caatinga currently faces increasing threats due to the exposure of its forest remnants to multiple anthropogenic pressures, such as agricultural expansion, deforestation, and an accelerated process of desertification, as well as scientific neglect compared to other tropical forests (Hughes et al., 2013; Ribeiro et al., 2015; Vieira et al., 2021). In this context, even though it houses more than 27 million people, it is observed that the Caatinga is one of the least studied global ecological regions, highlighting the urgent need for conservation and ongoing research to understand and protect its physiognomies (Lessa et al., 2019).

Understanding plant phenology is a fundamental aspect of ecology, providing insights into both community dynamics and the temporal fluctuations in resources available to consumers (Williams et al., 1999). Essentially, the phenological study of plants refers to the investigation of vegetative and reproductive development patterns throughout the year (Lieth, 1975). In recent decades, this type of study has provided information about ecosystem responses to climate change (Rubim et al., 2010). The recent progress in research in this field has also contributed to making phenology a predictive science (Piao et al., 2019).

Different phenological strategies have been reported in seasonally dry tropical forests (García-Cervigón et al., 2020; Silva et al., 2020). However, given the uniqueness of the Caatinga and the lack of scientific contributions, understanding ecological processes, such as those related to the growth and reproduction of plant species, can inform planning and forest management actions, enabling the establishment of a connection between the exploitation and conservation of natural resources.

The biometrics of fruits and seeds, in turn, are a characteristic that facilitates classification into different ecological groups. This approach can serve as a tool to identify successional strata in the Caatinga (Patrício and Trovão, 2020). Seeds of species found in the early stages of succession exhibit larger sizes. This simplifies the establishment process in the environment, given the absence of competitors and a broader nutritional reserve for the embryo (Patrício and Trovão, 2020).

The *Sarcomphalus joazeiro* (Mart.), an evergreen species endemic in the Northeast region, belongs to the Rhamnaceae family. The juazeiro, as it is popularly known in the region, occurs in the Caatinga biome and transition zones. It is utilized for various purposes, ranging from its application in traditional medicine (Lucas et al., 2021; Araújo et al., 2022) to its use in animal feed – especially during dry periods – in the production of cosmetics and personal hygiene products, in pharmacology and veterinary medicine, and for reforestation, providing thermal comfort due to its broad canopy (Santos et al., 2023). Moreover, *S. joazeiro* plays an essential role in the ecosystem of the region, providing pollen and nectar to native bees of the Meliponini tribe (Santos et al., 2023).

For these reasons, in this research, phenological observation of *Sarcomphalus joazeiro* (Mart.) was conducted to identify the ecological strategies adopted by the species to ensure success amidst a drought. To achieve this objective, the following question were answered: i) are reproductive phenophases strongly correlated with meteorological variables? ii) does the species exhibit intra-populational synchrony and an aggregated distribution pattern for reproductive events?

2. Material and Methods

2.1. Target species

Sarcomphalus joazeiro Mart. exhibits a wide distribution and usage in Brazil, standing out for its ornamental qualities and economic importance in forestry, food, and medicine, especially for traditional communities in the northeastern region of the country (Araújo, 2015;

Oliveira et al., 2017). With individuals reaching up to 16 meters in height and displaying a wide crown, this species has tri-nerve leaves, globose to ellipsoid drupes, and greenish-yellow flowers arranged in terminal or auxiliary inflorescences (Barroso et al., 1991).

It is a promising natural product in the development of phytotherapeutics; its extract demonstrates antifungal, antibacterial, and antiparasitic activity (Andrade et al., 2019a, b). Traditional communities report the use of the species for oral treatments (seborrhea and gingivitis), and research confirms its antioxidant activity, making it suitable for dental procedures, including acting as a tooth enamel whitener and protector (Rego, 2019; Fernandes and Bizerra, 2020). Leaves and bark shavings are used in detergents, shampoos, and other hair care and cosmetic products due to their richness in saponins composed of bioactives that exhibit anti-helminthic effects (Gomes et al., 2016). It is a species with multiple purposes and has been extensively researched for its medicinal applications, although there are few studies on its ecology and conservation (Duarte et al., 2018).

2.2. Study area

The research was conducted in a fragment of Seasonal Deciduous Forest, as classified by the Brazilian Institute of Geography and Statistics (IBGE, 2012), named Mata do Olho d'Água, located in the municipality of Macaíba, Northeast region of Brazil, between the coordinates 5°53'07.1" S and 35°22'02.6" W, with an average elevation of 40 meters above sea level and an area of approximately 270 hectares. The local forest represents a transition area between the Atlantic Forest and the Caatinga, both floristically and environmentally (Cestaro and Soares, 2004). The climate is characterized as a transition between the As' and BSh' types according to the Köppen classification (Alvares et al., 2013), with an average temperature of 25.8 °C and an annual average rainfall of 1,134 mm, marked by high temperatures throughout the year with rainfall in autumn and winter (Fig. 1).

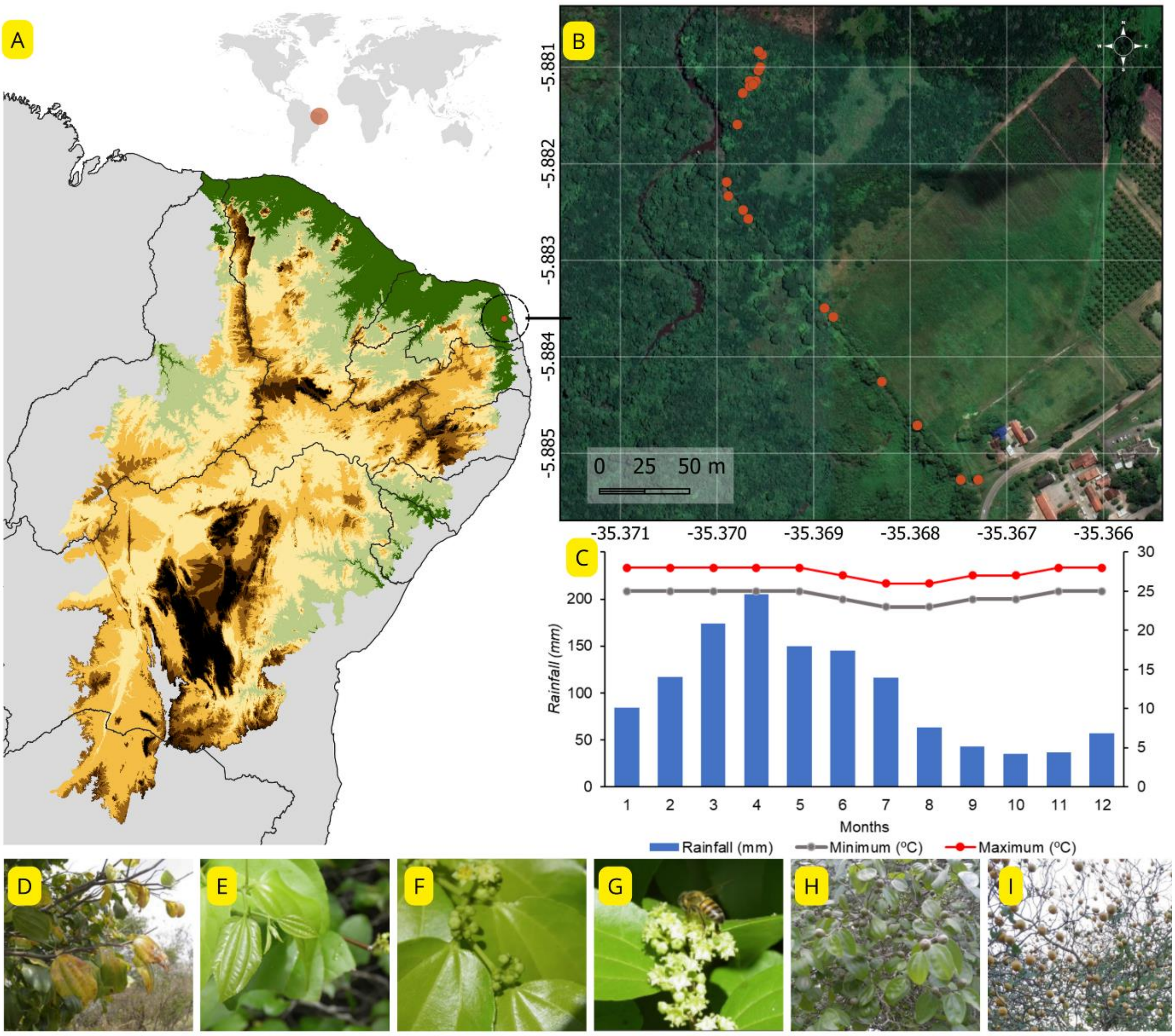

Figure 1. Distribution of the Caatinga among Brazilian states (A); *Sarcomphalus joazeiro* population used in the study (B); historical average of monthly precipitation values (bars) and minimum and maximum temperatures for the municipality of Macaíba – RN (C); phenophases: defoliation (D); leaf budding (E); flower buds (F); flowers anthesis (G); immature fruits (H) and ripe fruits (I).

2.3. Phenological data collection

Twenty individuals constituting a population of *S. joazeiro* were sampled. The selection criterion was determined by the presence of reproductive events and good phytosanitary conditions (apparent absence of diseases and parasite infestations). The sampling method for the population was the trail method (d'Eça-Neves and Morellato, 2004), considering that the individuals were arranged along an existing access path. Half of the individuals were located at the edge, while the others were under the influence of competition in a zone of moderately dense vegetation.

A total of 54 observations were carried out every two weeks between June 2016 and July 2018, with the monthly average of observations being calculated at the end of each month. We

evaluated the vegetative phenophases of leaf budding and defoliation, along with the reproductive phenophases of the presence of floral buds, flowers anthesis, immature fruits, and ripe fruits.

The assessment method employed was the Fournier index, a semi-quantitative method that determines the intensity at which the phenophase occurs. This method evaluates phenophases on a constant interval scale with five occurrence intensities ranging from 0 to 4. A value of zero represents the absence of the phenophase occurrence; 1 represents the occurrence of the phenophase with an intensity of 1 to 25% canopy coverage; value 2 represents the occurrence with an intensity of 26 to 50%; value 3 represents the occurrence within an interval of 51 to 75%; and value 4 represents the occurrence with 76 to 100% canopy coverage (Fournier, 1974).

For each conducted observation, the sum of intensity values for all individuals was calculated separately for each phenophase. Subsequently, it was divided by the total number of evaluated individuals (N = 20). This result was then multiplied by 100 to transform it into a percentage ($F\% = (\sum \text{intensities}/4N) * 100$) (d'Eça-Neves and Morellato, 2004).

2.4. Seasonality of phenophases

To identify the seasonality of the species, we conducted circular statistical analyses using the demo program Oriana® version 4.02 (available at https://www.kovcomp.co.uk/oriana/index.html). For this purpose, we converted an average date for each month into angles of the Julian calendar. We conducted the Rayleigh test for each phenological stage and calculated the mean vector length (r). This test assesses whether there is uniformity in the data distribution throughout the year. If the Rayleigh test indicates non-uniformity ($p < 0.01$), we interpret the value of r for each phenophase as a measure of seasonal intensity. The r vector indicates the degree of aggregation near the mean angle, ranging from 0 to 1, where higher values reflect greater seasonality (Morellato et al., 2010). Additionally, the angle representing the mean date of phenophase intensity (mean angle – μ) was converted. Furthermore, we employed the Watson-Williams test to compare results between the two assessment years ($p < 0.01$).

2.5. Spatial distribution of reproductive synchrony

The spatial distribution of flower and fruit events was analyzed based on data from a reproductive date, in other words, an observation that recorded high occurrence and intensity of phenophases in the population. For this purpose, we collected the coordinates of all trees that

exhibited flowering or fruiting events on that specific date. The neighborhood density with the same intensity of reproductive events was estimated using Moran's I index with the assistance of the ROOKCASE program (Sawada, 1999), along with exact Monte Carlo tests to assess the significance of autocorrelation values. We constructed a correlogram illustrating autocorrelation for different distance classes of mapped reproductive phenophases, enabling the evaluation of the proximity effect between sets of sample pairs.

2.6. Correlation with meteorological variables

The relationships between the occurrence of each phenophase and the air temperature, precipitation, and relative humidity variables were tested using Spearman's correlation. The correlation was performed with climatic data from the first (r_{S1}), second (r_{S2}), third (r_{S3}), and fourth (r_{S4}) fortnights preceding the observation of phenological events. For the statistical analysis of the data, deviations from the normality of phenological data were assessed using the Lilliefors test to justify the non-parametric Spearman correlation. Meteorological data were obtained from the climatological automatic station of the National Institute of Meteorology (INMET) closest to the study area, located in the municipality of Natal, approximately 20 km away.

2.7. Biometry of fruits and seeds

For biometric characterization, 200 ripe fruits were randomly collected from 5 trees in the population that were not monitored by phenology. The evaluated characteristics included fresh weight (g), length (mm), diameter (mm), and thickness (mm). After measurement, the fruits were depulped, and the same characterization was conducted for the seeds. Dimensions were determined using a digital caliper (precision of 2 mm), and fresh weight was determined using a precision analytical balance. Descriptive statistics were performed for the obtained data.

3. Results

3.1. Phenology and seasonality

By observing the phenophases of *S. joazeiro* over the 24 months, important results were obtained that can help in defining management strategies for the species. the Watson-Williams test showed a significant difference in the distribution of phenological events between the two evaluation years; however, the mean date of occurrence was preserved for most phenophases (Table 1).

Table 1. Statistical description of phenology and occurrence of seasonality for the *Sarcomphalus joazeiro* Mart. population. Average angle (μ), number of observations of phenophase manifestation (N), Rayleigh tests (Z) to verify seasonality ($p < 0.01$), intensity vector r, and Watson-William's test between the years of observation ($p < 0.01$). $Year_1$ = 2016/2017 and $Year_2$ = 2017/2018.

Phenophase	Year	μ	Mean date	N	Z	r	Watson-Williams test
Defoliation	$Year_1$	211.192°	August	383	< 0.001	0.158	< 0.001
	$Year_2$				ns*	-	
Leaf budding	$Year_1$	310.107°	November	137	< 0.001	0.408	< 0.001
	$Year_2$	342.591°	December	316	< 0.001	0.248	
Flower buds	$Year_1$	330.685°	December	68	< 0.001	0.856	< 0.001
	$Year_2$	352.942°	December	166	< 0.001	0.845	
flowers anthesis	$Year_1$	340.305°	December	96	< 0.001	0.849	< 0.001
	$Year_2$	356.921°	December	167	< 0.001	0.827	
Green fruits	$Year_1$	43.916°	February	224	< 0.001	0.778	< 0.001
	$Year_2$	73.355°	March	284	< 0.001	0.622	
Ripe fruits	$Year_1$						ns*
	$Year_2$						

*ns = not significant (p > 0,01)

The defoliation of *S. joazeiro* occurred throughout the study period, albeit with reduced intensity in most months (below 50%) (Fig. 2). The species exhibited low or no seasonality between the evaluated years (Table 1). The month with the highest intensity was October, with the observed mean date in August. On the other hand, leaf flush showed seasonality in both years, albeit low r values ($r_1 = 0.408$, $r_2 = 0.248$). Peaks of new leaf production mainly occurred during the dry period, with mean dates occurring between November and December.

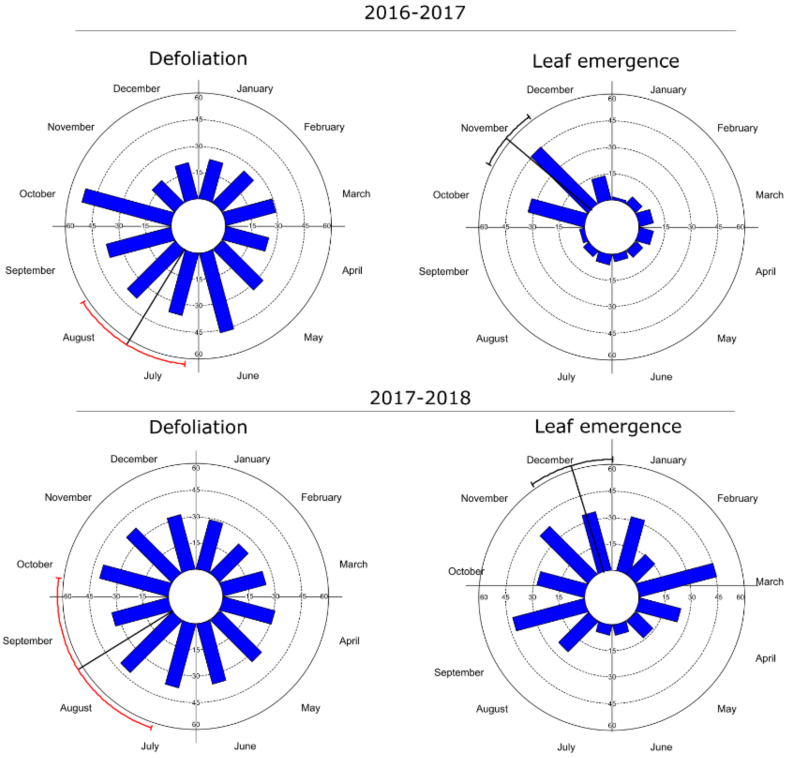

Figure 2. Histograms of the intensity of vegetative phenophases (leaf budding and defoliation) of *Sarcomphalus joazeiro* Mart. over the months. The arrow points to the average angle (or mean date) of the manifestation of the phenological event.

All reproductive events of *S. joazeiro* exhibit seasonality. Floral buds and flower production occurred simultaneously, beginning in the dry season, with December as the mean date (Fig. 3). Additionally, the studied population shows high synchrony in reproductive events, as the activities were recorded simultaneously in 80% of the individuals.

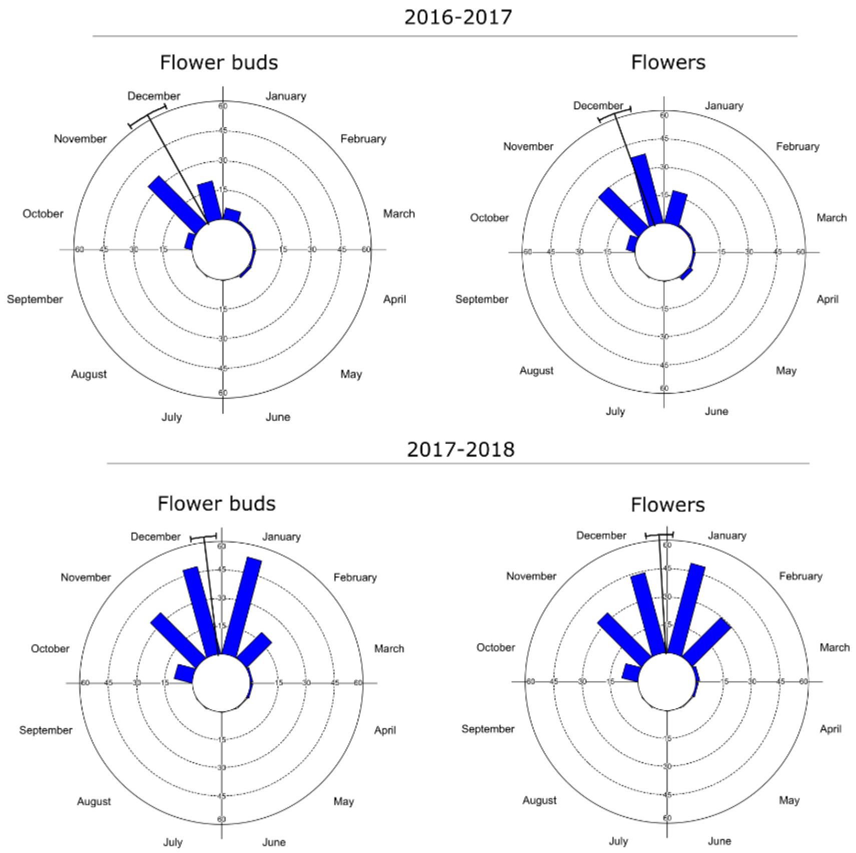

Figure 3. Histograms of intensity portraying the flowering (floral buds and flowers) of *Sarcomphalus joazeiro* Mart. across the months. The arrow indicates the average angle (or mean date) of the manifestation of the phenological event.

Fruit production also occurred seasonally. Immature fruits were intensively produced starting in December, with February and March as the mean observation dates (Fig. 4). Maturation followed soon after, with the mean date in April, representing the most suitable period for collection. During the second year, the occurrence of ripe fruits extended frequently, albeit at low intensity, throughout almost all months.

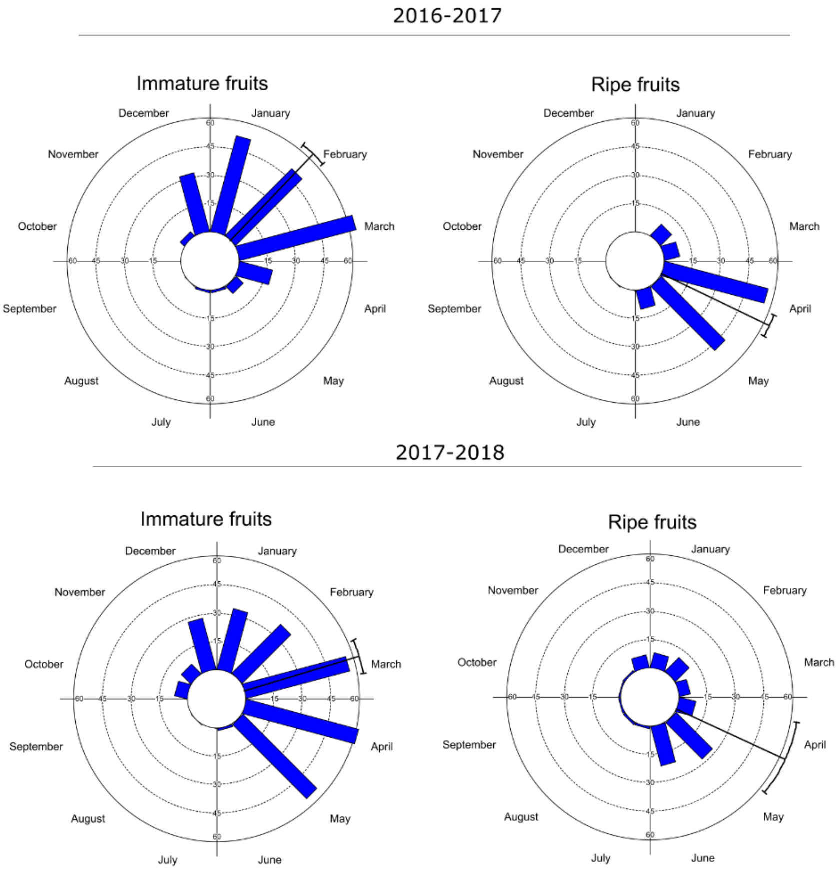

Figure 4. Histograms of the intensity of fruiting (immature and ripe fruits) of *Sarcomphalus joazeiro* over the months. The arrow points to the average angle (or mean date) of the manifestation of the phenological event.

3.2. Spatial distribution of reproductive synchrony

According to the Moran's I index value (Fig. 5), plants with higher flowering intensity exhibited a spatially aggregated pattern up to 36 meters (Moran's I = 0.290, p = 0.028, dashed line), as did fruiting (Moran's I = 0.527, p = 0.026, solid line). Flowering intensity increased between the classes of 92 to 104 meters but not observed for fruiting.

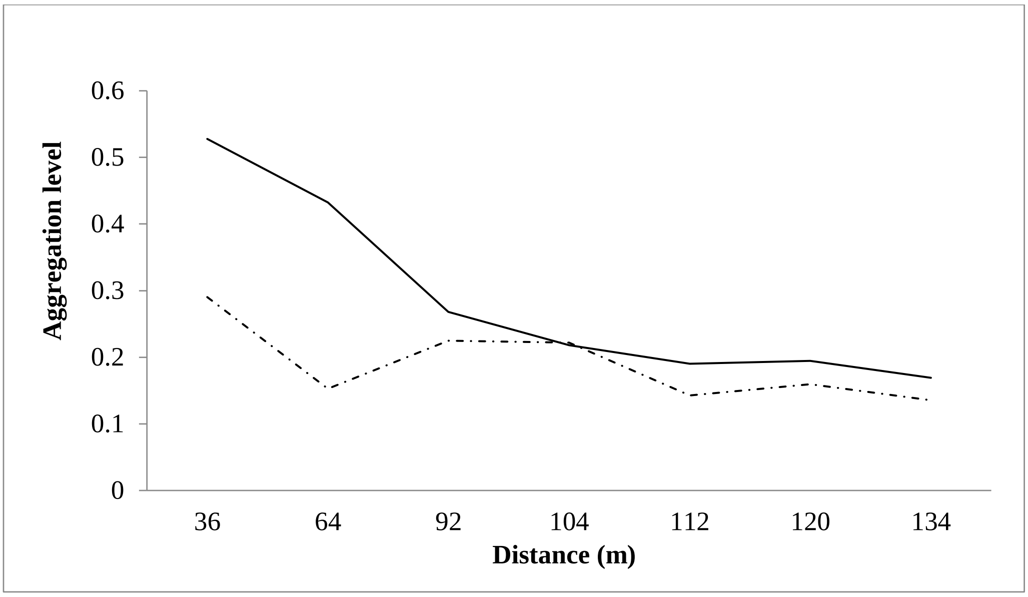

Figure 5. Moran's I correlogram illustrating the aggregated spatial pattern among individuals with flowering (---) and fruiting (—) events in the first distance classes.

3.3. Correlation with meteorological variables

Sarcomphalus joazeiro exhibited weak or no correlation with meteorological variables, indicating that air temperature, relative humidity, and precipitation had little influence on phenophases during the evaluated period.

Table 2. Significant Spearman correlation (rS) between the fortnightly means of climatic variables and the vegetative and reproductive phenological events of *Sarcomphalus joazeiro* Mart.

**Variables/fortnights***		**Defoliantion**	**Leaf budding**	**Flower buds**	Flowers **anthesis**	**Immature fruits**	**Ripe fruits**
**Air temperature** (°C)	r_{s1}	-0.552	ns	0.175	0.544	0.408	ns
	r_{s2}	-0.592	ns	0.374	0.514	0.501	ns
	r_{s3}	-0.547	ns	0.329	0.436	0.550	0.289
	r_{s4}	-0.510	ns	0.175	0.282	0.571	0.401
**Relative humidity** (%)	r_{s1}	ns	ns	-0.514	-0.498	ns	0.452
	r_{s2}	0.286	ns	-0.553	-0.554	ns	0.346
	r_{s3}	0.496	ns	-0.647	-0.641	ns	0.022
	r_{s4}	0.535	ns	-0.553	-0.603	-0.357	ns
**Precipitation** (mm)	r_{s1}	ns	ns	-0.279	ns	ns	ns
	r_{s2}	ns	ns	-0.365	-0.277	ns	ns
	r_{s3}	ns	ns	-0.365	-0.335	ns	ns
	r_{s4}	0.284	ns	-0.346	-0.348	ns	ns

*Correlations of the first (r_{S1}), second (r_{S2}), third (r_{S3}) and forth (r_{S4}) fortnights prior to the phenological event. ns: correlation not significant ($p > 0.05$)

3.4. Biometry of fruits and seeds

The biometric data for *S. joazeiro* fruits were 2.73 g, 16.94 mm, 14.52 mm, and 16.34 mm for fresh weight, diameter, length, and thickness, respectively. Meanwhile, the average data for seeds were 0.41 g, 7.55 mm, 11.00 mm, and 7.11 mm for the characteristics mentioned (Table 3).

Table 3. Means of biometric characteristics of fruits and seeds of *Sarcomphalus joazeiro*. N: sample size, CV: coefficient of variation, standard deviation, and mean.

Biometric characteristics		N	Maximum	Minimum	Mean ± standard error	Standard deviation	CV (%)
Fruit	mass (g)	200	4.25	1.30	2.73±0.04	0.63	23.32
	diameter (mm)	200	20.10	12.10	16.94±0.12	1.64	9.69
	lenght (mm)	200	17.80	11.60	14.52±0.08	1.23	8.46
	thickness (mm)	200	19.70	12.00	16.34±0.12	1.68	10.27
Seed	mass (g)	200	0.20	0.08	0.41±0.01	0.10	24.90
	diameter (mm)	200	9.40	5.50	7.55±0.05	0.76	10.17
	lenght (mm)	200	13.10	8.00	11.00±0.05	0.80	7.13
	thickness (mm)	200	9.00	5.00	7.11±0.05	0.70	9.74

4. Discussion

Phenological studies of species in the seasonal dry tropical forest (SDTF) are important to understand the patterns of flowering, fruiting, and other phases of the plant life cycle due to the marked variations between dry and rainy periods. However, the response of trees to drought in SDTF is complex. Species exhibit a range of strategies concerning leaf production and shedding, spanning the spectrum from deciduous to evergreen (Valdez-Hernández et al., 2010), which can affect the functional diversity of leaf traits due to aridity (Oliveira et al., 2020).

In some cases, underground water reserves and physiological adaptations mitigate the effects of rainfall scarcity, making phenological patterns weakly linked to environmental variables. This phenomenon is reflected in the vegetative phases of *S. joazeiro*. The species is considered evergreen with seasonal renewal (Pirani et al., 2009), given that it produces new leaves before or concurrently with the loss of old leaves. Thus, despite a significant percentage of leaf area loss in the canopy, the species never becomes completely leafless, as leaf budding

occurs simultaneously with the shedding of old leaves. This leaf exchange and flowering during the drought in *S. joazeiro* result from a series of mechanisms: efficient stomatal performance, coupled with the composition of its leaf epicuticular wax, characterized by a chemical constitution highly effective against water permeability (Oliveira et al., 2003). Additionally, the extensive root system with deep radial roots and resilient hydraulic architecture (smaller vessel diameters and stronger vessel walls) can tolerate significant soil moisture limitations (Wright et al., 2021).

In this sense, water availability in dry forests significantly influences the development of certain plant species more than the precipitation volume. Some studies demonstrate the high drought tolerance of *S. joazeiro* (Trovão et al., 2007). These results contribute to the explanation by Borchert and Rivera (2001), who state that species with mechanisms for more efficient water acquisition or storage may exhibit phenological patterns independent of rainfall rates.

Thus, it was demonstrated that there is a low or no significant correlation of the species' phenology with precipitation, temperature, and relative air humidity, unlike the species *Geoffrea spinosa*, which, in a phenological study in the same fragment, showed correlations of meteorological variables with some phenophases (Costa et al., 2022). This factor is interesting to understand because the species has different characteristics from other species in the Caatinga, as many species lose their leaves during drier periods, but this does not occur with *S. joazeiro*, possibly indicating adaptability to the region. This pattern suggests that it may be necessary to evaluate the species' interaction with the abiotic environment differently. It reinforces how environmental behavior varies according to the studied species and its ecological mechanisms.

The *S. joazeiro* flower and fruit production are annual, with the synchrony and the flowering period observed as reproductive strategies. Populations that exhibit synchrony during flowering may attract more pollinators and enhance pollen flow, leading to increased fruit formation that contributes to the ecological success of the species (Augspurger 1981; Tachiki et al., 2010). The intensification of flowering between the classes of 92 to 104 meters may indicate that the species invested in a high intensity of flowers to achieve more significant pollination. However, the spatial distance between plants may have been less efficient an attractive for vectors, as there was a decrease in the fruiting rate in plants that were spatially more distant.

In the case of this population of *S. joazeiro*, it can be asserted that the formation of flowers and, consequently, fruits occur in clusters. This is an important characteristic that contributes to its reproductive strategy, as the species enhances the floral display, and combined with

osmophores and pigments that reflect ultraviolet rays, they constitute an attractive ensemble for pollinators (Souza, 2016).

The fruiting period in the fragment coincides with what is observed in other regions, with ripe fruits available for collection in the first half of the year (January to July) (Oliveira et al., 2020). This information is crucial for guiding the seed collection period for seedling production, which can be implemented for various purposes. However, the production of ripe fruits practically every month in the second year likely occurred due to disruptions stemming from anthropogenic pressures in the area, which may have affected maturation synchrony.

Regarding the interaction with fauna, Barbosa et al. (1989), studying phenological data of 10 tree species in the Caatinga, observed that *S. joazeiro* is one of the few species that flowers during the dry season, potentially serving as an essential food resource for fauna during the drought. Its flowers are primarily visited by Hymenoptera from the families Apidae, Vespidae, Crabronidae, Leucospidae, and by Diptera from the families Syrphidae, Muscidae, Calliphoridae, Tabanidae, Dolichopodidae, Stratiomydae, Otitidae, and Micropezidae, with nectar being the main floral resource sought by the majority of visitors (Nadia et al., 2007; Leite and Machado, 2010; Fernandes et al., 2013).

Sarcomphalus joazeiro exhibits a syndrome of zoochorous dispersal; its fruits ripen and become available for consumption during the rainy season, reaching the average occurrence date in April. Although it is a species highly tolerant to the lack of rain, precipitation can also favor fruit production (Silva et al., 2020). The highest number of frugivorous birds are present in the Caatinga during increased rainfall, with *S. joazeiro* serving as a food source. The mammals *Mazama gouazoubira* and *Sapajus libidinosus* have been identified as potential dispersers (Silva et al., 2020).

Studying the biometrics of fruits and seeds from different *S. joazeiro* matrices, Lucena et al. (2017) found similar values to ours, observing fruits with an average length of 13.8 cm and a diameter of 18.6 cm, while seeds had a length of 10.6 cm and a diameter of 6.9 cm. The obtained results show that *S. joazeiro* seeds are larger than those of other coexisting species in the same community, such as *Mimosa tenuiflora* (Willd.) Poir. (Walter et al., 2019), and *Pityrocarpa moniliformis* (Benth.) Luckow & R. W. Jobson (Felix et al., 2020), all of these are pioneer species, whose main characteristic is the production of small seeds. Biometric analysis provides valuable information beyond the mere characterization of the species. With these data, helpful information for conservation, interspecific differentiation, economic resource exploitation, genetic variability among populations, and genetic improvement can be observed,

so the analysis of pulp yield from fruits is important both for fresh consumption and processing (Carvalho et al., 2003; Felix et al., 2020).

In this sense, it is emphasized that the protecting of various plant species is essential for future effective conservation strategies in the Caatinga, ensuring a constant presence of fruits (Silva et al., 2020). These fruits provide propagation availability and the possibility of individual entry into the areas. Moreover, fleshy fruits may be a food source for wildlife, which is even more crucial during drought and food scarcity (Leal et al., 2018).

5. Conclusions

The study provided valuable insights into the dynamics of *S. joazeiro* and the strategies adopted for its ecological success. It was possible to identify the seasonality present in its reproductive events and how changes in phenological dynamics can affect the biodiversity dependent on its resources.

The species exhibits peculiarities that make it stand out among SDTF trees. Its mechanisms render it resistant to the lack of rainfall, so its phenological performance is weakly correlated with meteorological variables. It is characterized as evergreen with seasonal renewal, featuring floral production during the dry season and fruit production at the beginning of the rainy period.

Overall, the study of the ecological processes of *S. joazeiro* contributes to understanding the complex dynamics of STDF. Conducting phenological studies with other species is a fundamental step to anticipate and mitigate the impacts of climate change in these regions, which are particularly vulnerable to environmental alterations.

Studying the dynamics of plants, the response of fauna, and the complex interactions between biotic and abiotic components of these forests contributes not only to scientific knowledge but also to the development of effective conservation strategies.

CRediT authorship contribution statement

Fernanda M. F. Lucas: Writing – review & editing, Writing – original draft, Formal analysis, Conceptualization, Visualization, Software, Methodology, Investigation, Formal analysis, Data curation**. Kyvia P. T. das Chagas:** Writing – review & editing, Writing – original draft, Formal analysis, Conceptualization, Visualization, Software, Methodology, Investigation, Formal analysis, Data curation. **Ageu S. M. Freire:** Writing – review & editing, Writing – original draft, Formal analysis, Conceptualization, Visualization, Software,

Methodology, Investigation, Formal analysis, Data curation **Vivian R. B. de Sousa:** Writing – review & editing, Conceptualization, Visualization. **Fábio A. Vieira:** Writing – review & editing, Supervision, Funding acquisition, Validation, Project administration,

Data Availability

The datasets produced and examined during the present investigation are freely available within the manuscript and can be obtained from the corresponding author upon a reasonable request.

Funding

This work was financed in part by the Office to Coordinate Improvement of Higher Education Personnel - Brazil (CAPES) - Finance Code 001. The authors acknowledge the Conselho Nacional de Desenvolvimento Científico e Tecnológico (CNPq) for their financial support (Grant no. 407700/2023-4).

Declaration of competing interest

The authors declare they have no conflicts of interest.

References

Alvares CA, Stape JL, Sentelhas PC, et al (2013) Köppen's climate classification map for Brazil. Meteorologische Zeitschrift 22:711–728. https://doi.org/10.1127/0941-2948/2013/0507

Andrade JC, Pereira Da Silva AR, Thassya A, et al (2019a) Chemical composition, antiparasitic and cytotoxic activities of aqueous extracts of *Ziziphus joazeiro* Mart. https://doi.org/10.4103/2221-1691.259003

Andrade JC, Silva ARP, Audilene Freitas M, et al (2019b) Control of bacterial and fungal biofilms by natural products of *Ziziphus joazeiro* Mart. (Rhamnaceae). Comp Immunol Microbiol Infect Dis 65:226–233. https://doi.org/10.1016/J.CIMID.2019.06.006

Araújo BA, Silva MCB, Moreira FJC, et al (2015) Biometric Characterization of fruits and seeds, chemical and pulp yield of juazeiro (*Ziziphus joaseiro* Mart.). Revista agropecuária científica no semiárido 11(2), 15-21. https://doi.org/10.30969/acsa.v11i2.605

Araújo NJS, Silva ARP, Costa MS, Freitas TS, Filho JMB, Matos YMLS, Morais-Braga MFB, Junior FNP, Silva CAP, Souza EO, Ribeiro PRV, Lacerda BCGV, Andrade EM, Coutinho HDM, Pinheiro JCA. Chemical characterization UPLC-ESI-QToF-MSE, antibacterial and antibiofilm potential of *Sarcomphalus joazeiro* (MART.) Hauenschild, Food Bioscience, Volume 50, Part A, 2022, 102066. https://doi.org/10.1016/j.fbio.2022.102066

Augspurger CK (1981) Reproductive Synchrony of a Tropical Shrub: Experimental Studies on Effects of Pollinators and Seed Predators in *Hybanthus prunifolius* (Violaceae). Ecology 62:775–788. https://doi.org/10.2307/1937745

Barbosa DC de A, Alves JL de H, Prazeres S de M, Paiva AMA (1989) Dados fenológicos de 10 espécies arbóreas de uma área de caatinga (Alagoinha-PE). Acta Bot Brasilica 3:109–117. https://doi.org/10.1590/S0102-33061989000300011

Barroso GM, Peixoto AL, Ichaso CLF, Costa CG, Guimarães EF, Lima HC (1991) Sistemática de angiospermas do Brasil, v.2. Editora UFV: Viçosa, 1991. ISBN 8572691278

Borchert R, Rivera G (2001) Photoperiodic control of seasonal development and dormancy in tropical stem-succulent trees. Tree Physiol 21:213–221. https://doi.org/10.1093/treephys/21.4.213

Carvalho JEU de, Nazaré RFR de, Nascimento WMO do (2003) Physical and physicochemical characteristics of a bacuri (*Platonia insignis* Mart.) type with better industrial yield. Revista Brasileira de Fruticultura. 25:326–328. https://doi.org/10.1590/S0100-29452003000200036

Cestaro LA, Soares JJ (2004) Floristic and structural variations, and the phytogeographical relationships of a deciduous forest fragment in Rio Grande do Norte State, Brazil. Acta Bot Brasilica 18:203–208. https://doi.org/10.1590/S0102-33062004000200001

Oliveira PM de L, Dantas AM, Morais AR dos S, et al (2020) Juá fruit (*Ziziphus joazeiro*) from Caatinga: A source of dietary fiber and bioaccessible flavanols. Food Research International 129:108745. https://doi.org/10.1016/J.FOODRES.2019.108745

d'Eça-Neves FF, Morellato LPC (2004) Methods applied for sampling and estimate tropical forest phenology. Acta Bot Brasilica 18:99–108. https://doi.org/10.1590/S0102-33062004000100009

Duarte MM, Nogueira AC, Vieira ESN (2018) Diversity and spatial genetic structure of natural populations of *Ziziphus joazeiro* Mart. Revista Brasileira de Ciências Agrárias - Brazilian Journal of Agricultural Sciences 13:1–6. https://doi.org/10.5039/agraria.v13i4a5573

Felix FC, Medeiros JAD, Ferrari CS, et al (2020) Biometry of *Pityrocarpa moniliformis* seeds using digital imaging: implications for studies of genetic divergence. Revista Brasileira de Ciências Agrárias - Brazilian Journal of Agricultural Sciences 15:1–8. https://doi.org/10.5039/agraria.v15i1a6128

Fernandes EC, Lima ADCF, Araújo EL, et al (2013) Insectos visitantes de flores de *Ziziphus joazeiro* Mart. (Rhamnaceae) en una región de Caatinga en el Brasil. Revista Caatinga 26:16–20

Fernandes MF, Cardoso D, Pennington RT, de Queiroz LP (2022) The Origins and Historical Assembly of the Brazilian Caatinga Seasonally Dry Tropical Forests. Front Ecol Evol 10:723286. https://doi.org/10.3389/FEVO.2022.723286/BIBTEX

Fernandes PRD, Bizerra AMC (2020) Quantitative evaluation of antioxidant activities of native plants of the Alto Oeste Potiguar Region/RN. Research, Society and Development 9:e48911578. https://doi.org/10.33448/rsd-v9i1.1578

Fournier, LA (1974) Un método cuantitativo para la medición de características fenológicas em árboles. Turrialba, 24:422–423.

García-Cervigón AI, Camarero JJ, Cueva E, et al (2020) Climate seasonality and tree growth strategies in a tropical dry forest. Journal of Vegetation Science 31:266–280. https://doi.org/10.1111/JVS.12840

Gomes DC, de Lima HG, Vaz AV, et al (2016) In vitro anthelmintic activity of the *Zizyphus joazeiro* bark against gastrointestinal nematodes of goats and its cytotoxicity on Vero cells. Vet Parasitol 226:10–16. https://doi.org/10.1016/J.VETPAR.2016.06.004

Hughes CE, Pennington RT, Antonelli A (2013) Neotropical Plant Evolution: Assembling the Big Picture. Botanical Journal of the Linnean Society 171:1–18. https://doi.org/10.1111/BOJ.12006

IBGE IB de G a e E (2012) Manual Técnico da Vegetação Brasileira, 2nd edn. Instituto Brasileiro de Geografia e Estatística, Rio de Janeiro, Brasil

Leal IR, Lopes A V., Machado IC, Tabarelli M (2018) Interações planta-animal na Caatinga: visão geral e perspectivas futuras. Cienc Cult 70:35–40. https://doi.org/10.21800/2317-66602018000400011

Leite AV de L, Machado IC (2010) Reproductive biology of woody species in Caatinga, a dry forest of northeastern Brazil. J Arid Environ 74:1374–1380. https://doi.org/10.1016/j.jaridenv.2010.05.029

Lessa T, Dos Santos JW, Correia RA, et al (2019) Known unknowns: Filling the gaps in scientific knowledge production in the Caatinga. PLoS One 14:e0219359. https://doi.org/10.1371/JOURNAL.PONE.0219359

Lieth H (1975) Phenology and Seasonality Modeling in Ecological Studies vol. 8. Soil Sci 120:461

Lucas FMF, Chagas KPT, Vieira FA (2021) Modeling the spatial distribution of a tropical dry forest tree facing climate change. Revista Brasileira de Ciências Agrárias, 16:e8856. https://doi.org/10.5039/agraria.v16i1a8856

Lucena EO de, Lúcio AMF da N, Bakke IA, et al (2017) Biometrics and physiological quality of juazeiro seeds (*Ziziphus joazeiro* Marth.) matrix of different semiarid Paraiba. Agropecuária Científica no Semiárido 13:275. https://doi.org/10.30969/acsa.v13i4.897

Morellato LPC, Alberti LF, Hudson IL (2010) Applications of Circular Statistics in Plant Phenology: a Case Studies Approach. In: Phenological Research. Springer Netherlands, Dordrecht, pp 339–359

Murphy PG, Lugo AE (2003) Ecology of Tropical Dry Forest. 67–88. https://doi.org/10.1146/ANNUREV.ES.17.110186.000435

Nadia T de L, Machado IC, Lopes AV (2007) Reproductive phenology and pollination system of *Ziziphus joazeiro* Mart. (Rhamnaceae): the role of *Apis mellifera* and autochthonous floral visitors as pollinators Acta Bot Brasilica 21:835–845. https://doi.org/10.1590/S0102-33062007000400008

Oliveira ACP, Nunes A, Rodrigues RG, Branquinho C (2020) The response of plant functional traits to aridity in a tropical dry forest. Science of The Total Environment 747:141177. https://doi.org/10.1016/j.scitotenv.2020.141177

Oliveira AFM, Meirelles ST, Salatino A (2003) Epicuticular waxes from caatinga and Cerrado species and their efficiency against water loss. An Acad Bras Cienc 75:431–439. https://doi.org/10.1590/S0001-37652003000400003

Oliveira MID, Medeiros Araújo MB, Nascimento LV, et al (2017) Seed health of *Crataeva tapia* and *Ziziphus joazeiro*. Revista Verde de Agroecologia e Desenvolvimento Sustentável 12:858–861. https://doi.org/10.18378/RVADS.V12I5.5571

Patrício M da C, Trovão DM de BM (2020) Seed biometry: another functional trait in Caatinga. Acta Sci Biol Sci 42:e51183. https://doi.org/10.4025/actascibiolsci.v42i1.51183

Piao S, Liu Q, Chen A, et al (2019) Plant phenology and global climate change: Current progresses and challenges. Glob Chang Biol 25:1922–1940. https://doi.org/10.1111/GCB.14619

Pirani FR, Sanchez M, Pedroni F (2009) Phenology of a tree community in a cerrado sensu stricto, Barra do Garças, Mato Grosso State, Brasil. Acta Bot Brasilica 23:1096–1110. https://doi.org/10.1590/S0102-33062009000400019

Queiroz LP, Cardoso D, Fernandes MF, Moro MF (2018) Diversity and evolution of flowering plants of the Caatinga Domain. Caatinga: The Largest Tropical Dry Forest Region in South America 23–63. https://doi.org/10.1007/978-3-319-68339-3_2/COVER

Rego EL (2019) Ethnobotanic study on the use of júa *Ziziphus joazeiro* Mart. (Rhamnaceae) in the Serra Verde -PE community. Brazilian Journal of Health Review 2:5801–5810. https://doi.org/10.34119/bjhrv2n6-074

Ribeiro EMS, Arroyo-Rodríguez V, Santos BA, et al (2015) Chronic anthropogenic disturbance drives the biological impoverishment of the Brazilian Caatinga vegetation. Journal of Applied Ecology 52:611–620. https://doi.org/10.1111/1365-2664.12420

Rubim P, Nascimento HEM, Morellato LPC. Interannual variation in the phenology of a tree community in a semideciduous seasonal forest in southeast Brazil. Acta Botanica Brasilica, v. 24, n. 3, p. 756-762, 2010. https://doi.org/10.1590/S0102-33062010000300019

Sawada M (1999) ROOKCASE: An Excel 97/2000 Visual Basic (VB) Add-in for Exploring Global and Local Spatial Autocorrelation. Bulletin of the Ecological Society of America 80:231–234

Santos MM, Arriel EF, Bastos PM., Freire ALO, Ferreira CD, Carmo FCA, Leite GV, França GM (2023). Size of mini-cuttings for the production of *Sarcomphalus joazeiro* (Mart.) Hauenshild seedlings. Revista De Gestão E Secretariado, 14(9), 15716–15726. https://doi.org/10.7769/gesec.v14i9.2835

Silva AC, Souza AF (2018) Aridity drives plant biogeographical sub regions in the Caatinga, the largest tropical dry forest and woodland block in South America. PLoS One 13:e0196130. https://doi.org/10.1371/JOURNAL.PONE.0196130

Silva ÉE de M, Paixão VHF, Torquato JL, et al (2020) Fruiting phenology and consumption of zoochoric fruits by wild vertebrates in a seasonally dry tropical forest in the Brazilian Caatinga. Acta Oecologica 105:103553. https://doi.org/10.1016/J.ACTAO.2020.103553

Souza IP de (2016) Fenologia, biologia floral e dispersão de *Simarouba amara* Aubl. (Simaroubaceae) em Mata Ciliar na Chapada Diamantina, Bahia. Anais dos Seminários de Iniciação Científica. https://doi.org/10.13102/SEMIC.V0I20.3064

Tachiki Y, Iwasa Y, Satake A (2010) Pollinator coupling can induce synchronized flowering in different plant species. J Theor Biol 267:153–163. https://doi.org/10.1016/j.jtbi.2010.08.023

Trovão DMBM, Fernandes PD, De Andrade LA, Neto JD (2007) Seazonal variations of physiological aspects of Caatinga species. Revista Brasileira de Engenharia Agrícola e Ambiental 11:307–311. https://doi.org/10.1590/S1415-43662007000300010

Valdez-Hernández M, Andrade JL, Jackson PC, Rebolledo-Vieyra M (2010) Phenology of five tree species of a tropical dry forest in Yucatan, Mexico: Effects of environmental and physiological factors. Plant Soil 329:155–171. https://doi.org/10.1007/S11104-009-0142-7/FIGURES/5

Vieira RMDSP, Tomasella J, Barbosa AA, et al (2021) Desertification risk assessment in Northeast Brazil: Current trends and future scenarios. Land Degrad Dev 32:224–240. https://doi.org/10.1002/LDR.3681

Walter LS, Gabira MM, Nogueira AC (2019) Accelerated aging test in *Mimosa tenuiflora* (Willd.) Poir. seeds. Revista Forestal Mesoamericana Kurú 17:. https://doi.org/10.18845/rfmk.v17i40.4907

Williams RJ, Myers BA, Eamus D, Duff GA (1999) Reproductive Phenology of Woody Species in a North Australian Tropical Savanna. Biotropica 31:626–636. https://doi.org/10.1111/J.1744-7429.1999.TB00411.X

Wright CL, Lima ALA, Souza ES, et al (2021) Plant functional types broadly describe water use strategies in the Caatinga, a seasonally dry tropical forest in northeast Brazil. Ecol Evol 11:11808–11825. https://doi.org/10.1002/ece3.7949

Zappi DC, Ranzato Filardi FL, Leitman P, et al (2015) Growing knowledge: an overview of Seed Plant diversity in Brazil. Rodriguésia 66:1085–1113. https://doi.org/10.1590/2175-7860201566411
