# Supplementary material for: Phenological strategies of an evergreen tree in the Caatinga
Source: PLoS One. 2025 Mar 7;20(3):e0317522. doi: 10.1371/journal.pone.0317522 (PMC11888133; doi:10.1371/journal.pone.0317522)
Supplement: Attachment — Submitted filename: General comments on research paper.docx [file pone.0317522.s003.docx]

**General comments**

**Abstracts:** Abstract was not written well, thus try to refine it by adding the methodology and recommendation parts.

**Key words:** Capitalize the first letter of each word and add one more key word.

**Statement of the problem:** is not well stated; you stated that “to ensure success amidst a drought” but, the tree is already grown and growing in the area. Please write the main reason to study the functional strategies of *S. joazeiro* in a Brazilian Seasonally Dry Tropical Forest Fragment.

**Methodology:** the researchers used the presence of reproductive events and good phytosanitary conditions as tree selection criteria; but what about the effect of tree’s age on the phenology?

When you write the methodology of research article please, don’t personalize it. For example don’t use “we, I”

**Result and discussion:** written well

**Reference:** The reference writing style is not consistent. The researchers used et al in some parts of bibliography and not in others. I recommend writing the name of whole authors in bibliography. But it depends on the type of reference writing styles (Harvard, APA, MLA and others)
